# Frailty predicts outcome of partial nephrectomy and guides treatment decision towards active surveillance and tumor ablation

**DOI:** 10.1007/s00345-020-03556-7

**Published:** 2021-01-30

**Authors:** M. T. Walach, M. F. Wunderle, N. Haertel, J. K. Mühlbauer, K. F. Kowalewski, N. Wagener, N. Rathmann, M. C. Kriegmair

**Affiliations:** 1 Department of Urology and Urological Surgery, University Medical Center Mannheim, Heidelberg University, Theodor-Kutzer-Ufer 1-3, 68167 Mannheim, Germany; 2 Department of Medicine II, University Medical Centre Mannheim, Theodor‑Kutzer‑Ufer 1-3, 68167 Mannheim, Germany; 3 Department of Urology, Asklepios Klinik Altona, Paul-Ehrlich-Strasse 1, 22763 Hamburg, Germany; 4 Department of Radiology and Nuclear Medicine, University Medical Centre Mannheim, Theodor-Kutzer-Ufer 1-3, 68167 Mannheim, Germany

**Keywords:** Renal cancer, Nephron sparing surgery, Ablation, Active surveillance, Frailty, Geriatric assessment

## Abstract

**Purpose:**

To examine frailty and comorbidity as predictors of outcome of nephron sparing surgery (NSS) and as decision tools for identifying candidates for active surveillance (AS) or tumor ablation (TA).

**Methods:**

Frailty and comorbidity were assessed using the modified frailty index of the Canadian Study of Health and Aging (11-CSHA) and the age-adjusted Charlson-Comorbidity Index (aaCCI) as well as albumin and the radiological skeletal-muscle-index (SMI) in a cohort of *n* = 447 patients with localized renal masses. Renal tumor anatomy was classified according to the RENAL nephrometry system. Regression analyses were performed to assess predictors of surgical outcome of patients undergoing NSS as well as to identify possible influencing factors of patients undergoing alternative therapies (AS/TA).

**Results:**

Overall 409 patient underwent NSS while 38 received AS or TA. Patients undergoing TA/AS were more likely to be frail or comorbid compared to patients undergoing NSS (aaCCI: *p* < 0.001, 11-CSHA: p < 0.001). Gender and tumor complexity did not vary between patients of different treatment approach. 11-CSHA and aaCCI were identified as independent predictors of major postoperative complications (11-CSHA ≥ 0.27: OR = 3.6, *p = *0.001) and hospital re-admission (aaCCI ≥ 6: OR = 4.93, *p = *0.003) in the NSS cohort. No impact was found for albumin levels and SMI. An aaCCI > 6 and/or 11-CSHA ≥ 0.27 (OR = 9.19, p < 0.001), a solitary kidney (OR = 5.43, *p = *0.005) and hypoalbuminemia (OR = 4.6, *p = *0.009), but not tumor complexity, were decisive factors to undergo AS or TA rather than NSS.

**Conclusion:**

In patients with localized renal masses, frailty and comorbidity indices can be useful to predict surgical outcome and support decision-making towards AS or TA.

**Supplementary Information:**

The online version contains supplementary material available at 10.1007/s00345-020-03556-7.

## Introduction

Frailty and comorbidities in patients undergoing tumor surgery are a growing issue for surgeons as frail patients show increased mortality rates and hospitalizations [1]. Frailty is a common medical syndrome in older adults characterized by high risk for falls, diminished strength and endurance and overall reduced physiologic functions [2]. It can occur as a result of the accumulation of different medical conditions and diseases and can increase the vulnerability of developing a higher mortality rate when exposed to a stressor, such as surgery [3]. The relation of frailty syndrome, comorbidities, post-surgical outcome and overall-mortality is an important variable for the estimation of fitness for surgery and has recently emerged as an essential aspect for the estimation of perioperative risk in older patients across surgical interventions [4, 5].

In general, the frequency of surgical procedures has greatly increased in modern medicine and the ongoing increase in old-age population both contribute to the fact that surgery in elderly patients has become very common. This is relevant in diseases like kidney tumors, where active surveillance (AS) and ablative treatments can be an alternative treatment option to advanced surgical oncological interventions [6, 7]. Especially small renal masses (SRM) can be observed and watched closely within the AS concept without treatment at first to avoid the surgical intervention and possible associated complications in frail and comorbid patients [8, 9].

The exposure to stressors, such as surgery, may lead to a disproportional decompensation of the health state in frail patients suffering from kidney tumors. Therefore, the risk–benefit ratio should include an assessment for frailty in elderly patients planned to undergo nephron sparing surgery (NSS) [10].

We already know that the complexity of kidney tumors plays a crucial role for the postoperative outcome and can be measured using nephrometry scores [11]. The standardized categorization of renal masses supports surgical decision-making on the basis of tumor characteristics, namely the important anatomical aspects, without considering patient characteristics [12, 13]. Identifying influencing factors on the therapeutic strategy patients with renal cancer are advised to can support and affirm decision-making.

In this study, we evaluated the association of frailty and comorbidity parameters in patients undergoing NSS and the perioperative outcome to address the question whether patient characteristics should be given more attention before undergoing NSS. Furthermore, we assessed possible decisive and influencing factors to rather undergo AS or tumor ablation (TA) than NSS.

## Patients and methods

### Study design and data collection

The data collection for this study was approved by the local ethical committee. Ethical approval number for data acquisition is 2014-526N-MA.

We assessed patient and tumor characteristics in consecutive patients with renal masses at our high-volume university medical center between 01/2010 and 02/2020. 74 patients were excluded from the study for lack of informed consent, 61 patients for radiological missing data, four patients for conversion to radical nephrectomy (three of them because of pT2 tumor stage and one patient because of R1-resection). Our patient collective can be divided into two groups: one cohort comprising patients who underwent open or robotic NSS (*n* = 409) and another cohort consisting of patients who underwent AT or AS (*n* = 38) as treatment for kidney cancer. We only included patients older than 45 years in both cohorts. Data collection was performed using medical charts, laboratory investigations and radiological reports.

### Geriatric assessment

Geriatric and frailty parameters were assessed using different measurement tools. The primary tool to assess frailty was the modified frailty index of the Canadian Study of Health and Aging (11-CSHA), which is a validated tool based on clinical data and consisting of eleven elements. The sum score is divided by 11 and a cut-off of ≥ 0.27 has been defined to mirror frailty [14].

Another tool used to define comorbid patients was the age-adjusted Charlson Comorbidity Index (aaCCI). It includes different parameters, such as kidney, liver or pulmonary diseases, diabetes, dementia or congestive heart failure. The sum of the points is correlated to the mortality rate. We defined a cut-off of ≥ 6 points to represent frailty, according to the literature showing a cut-off range from 4 to 7 to mirror frailty [15]. Based on those assessment tools, patients were divided into frail and non-frail patients.

Moreover, albumin levels were assessed as hypoalbuminemia is thought to increase the risk of vulnerability and mortality. The skeletal index was assessed to measure sarcopenia by quantifying the lumbar skeletal muscle mass on the level of the third lumbar vertebra based on CT (computed tomography) or MRI (magnetic resonance imaging) scans, taken for diagnostic purposes (cut-off: ≤ 38.5 cm^2^/m^2^ for women and ≤ 52.4 cm^2^/m^2^ for men [16]).

### Tumor assessment and surgical outcome

To assess tumor characteristics, we collected tumor-specific data, such as tumor size and tumor complexity using the RENAL nephrometry score and information about solitary kidneys. Surgical outcome was assessed collecting data concerning operation time, ischemia time, blood loss, transfusion rate and TRIFECTA achievement (negative surgical margin, ischemia time < 25 min, no major complications) [17].

### Outcome measures

The primary outcome measures of this study were the impact of frailty on postoperative severe complications and hospital re-admission in patients undergoing NSS and the identification of factors influencing the therapeutic strategy, namely AT or AS, in patients with renal cancer. Postoperative complications were evaluated using the Clavien–Dindo classification (CDC, grades ≥ 3 representing severe complications) [18]. Complications and re-admission to the hospital referred to a 30-day period after surgery. Moreover, we assessed the transfusion rates and radiological tools, such as the skeletal muscle index, to measure sarcopenia. Furthermore, hypoalbuminemia (blood albumin level < 35 g/l) was assessed as it is thought to increase the risk of vulnerability and mortality. Surgical parameters, such as ischemia time, operation time and blood loss, were also correlated to frailty.

### Surgical technique

The operative approach in patients undergoing NSS was either open NSS, robot-assisted NSS using the da Vinci Xi^®^ surgical system or laparoscopic NSS performed via transperitoneal approach. The surgical technique was described in detail previously [19]. Patients who underwent an ablation procedure were either treated with microwave ablation (*n* = 6) or irreversible electroporation (*n* = 16). Ablation procedures were carried out by the interventional radiologists at our institution. Open Surgery was performed by 11 experienced surgeons, who have each already performed at least 100 open partial nephrectomies in our highly standardized technique. Likewise, robot-assisted partial nephrectomy was only performed by experienced surgeons. The procedural management was described in detail previously [20, 21].

### Statistical analysis

All statistical analyses were performed using statistical software JMP^®^ from SAS (version 13 for Windows, SAS Institute Inc.). For descriptive data with normal distribution, mean ± standard deviation (SD) was given. Comparisons between the groups were performed using the independent *t* test. A *p* value < 0.05 indicated statistical significance.

To analyze potential influencing and risk factors for severe complications and hospital re-admission rates and to evaluate influencing factors on the therapeutic strategy, uni- and multivariable logistic regressions were performed.

## Results

### Tumor and patient characteristics and surgical outcome in the cohorts

Overall, we assessed 409 patients in our NSS cohort and 38 patients in the AS/TA cohort. The patient and tumor characteristics in the two study groups are illustrated in Table 1. Patients in the AS/TA cohort were significantly older, had smaller tumours and more often a solitary kidney. In addition, the frailty and comorbidity scores 11-CSHA and aaCCI revealed higher scores in the AS/TA cohort, respectively. Notably, in the AS/TA group, only patients who underwent an intervention (*n* = 22) were considered addressing severe complications.

**Table 1:** Tumor and patient characteristics of the AS/TA cohort and the NSS cohort

	AS/TA cohort (*n* = 38)	NSS cohort (*n* = 409)	*p* value
Age > 70 years, *n* (%)	27 (71.1)	165 (40.3)	**0.005**
Male, *n* (%)	24 (63.2)	278 (68.0)	0.588
Tumor size (cm), mean ± SD	2.8 ± 1.2	3.5 ± 1.9	**0.041**
Solitary kidney, *n* (%)	11 (28.9)	26 (6.4)	**0.001**
RENAL, median (IQR)	8 (7–9)	7 (6–9)	0.512
11-CSHA, mean ± SD	0.31 ± 0.18	0.13 ± 0.11	** < 0.001**
aaCCI, median (IQR)	8 (6–10)	3 (2–4)	** < 0.001**
ASA score, median (IQR)	3 (2–3)	2 (2–3)	** < 0.001**
Clavien–Dindo (≥ 3), *n* (%)	2 (9.1)	57 (13.9)	0.753
Sarcopenia, *n* (%)	6 (15.8)	128 (31.3)	0.063
Hypoalbuminemia, *n* (%)	20 (52.6)	39 (9.5)	**0.001**

Bold font indicates statistical significance

The patient and tumor characteristics in the NSS subgroups frail and non-frail are illustrated in Table 2. 16.6% (*n* = 68) of the patients of the NSS cohort could be classified as frail. The proportion of patients older than 70 years was significantly larger in the frailty group. Furthermore, frail patients showed higher ASA scores and significantly more patients showed hypoalbuminemia.

**Table 2:** Tumor and patient characteristics of frail and non-frail patients according to the 11-CSHA in the NSS cohort

	No frailty (*n* = 341)	Frailty (*n* = 68)	*p* value
Age > 70 years, *n* (%)	118 (34.6)	47 (69.1)	** < 0.001**
Male, *n* (%)	228 (66.9)	50 (73.5)	0.321
Tumor size (cm), mean ± SD	3.5 ± 2	3.4 ± 1.4	0.607
Solitary kidney, *n* (%)	21 (6.2)	5 (7.4)	0.785
RENAL, median (IQR)	7 (7–9)	7 (5–9)	0.164
ASA score, median (IQR)	2 (2–2)	3 (2–3)	** < 0.001**
Sarcopenia, *n* (%)	99 (29.7)	29 (43.3)	0.148
Hypoalbuminemia, *n* (%)	28 (8.2)	11 (16.2)	**0.046**

Bold font indicates statistical significance

59 patients (13.7%) developed major postoperative complications (CDC ≥ 3). These were predominantly aneurysms requiring embolization (*n* = 22), pneumothoraces requiring thoracic drainage (*n* = 22), postoperative bleeding requiring surgical revision (*n* = 10) and anuria requiring transient dialysis (*n* = 7). Two patients died from massive postoperative bleeding.

The surgical outcome of non-frail and frail patients in the NSS cohort according to the 11-CSHA is shown in Table 3. There was no significant difference between the groups concerning ischemia time (18 vs. 19 min, *p = *0.926) and blood loss (310 vs. 313 ml, *p* = 0.953). The operation time (145 vs. 167 min, *p* = 0.002) and the transfusion rate (7.9 vs. 26.5%, *p* = 0.001) were significantly higher in the frailty group. Correspondingly, severe complications (Clavien–Dindo ≥ 3, 11.7 vs. 25%, *p* = 0.007), the length of the hospital stay (6 vs. 7 days, *p* < 0.001) and the re-admission rate (5.9 vs. 16.2%, *p* = 0.009) showed a significant difference between the two groups, to the disadvantage of the frailty group. TRIFECTA outcome did not differ significantly in non-frail compared to frail patients (49.9 vs. 36.8%, *p* = 0.062). Postoperative acute kidney injury (AKI) was higher in the frailty-group (51.6 vs. 66.2%, *p* = 0.033).

**Table 3:** Surgical outcome in frail patients compared to non-frail patients according to the 11-CSHA in the NSS cohort

	No frailty (*n* = 341)	Frailty (*n* = 68)	*p* value
Ischemia time (min), mean ± SD	18 ± 8	19 ± 10	0.926
Operation time (min), mean ± SD	145 ± 50	167 ± 61	**0.002**
Blood loss in (ml), mean ± SD	310 ± 376	313 ± 289	0.953
Transfusion, *n* (%)	27 (7.9)	18 (26.5)	**0.001**
TRIFECTA, *n* (%)	170 (49.9)	25 (36.8)	0.062
Clavien–Dindo (≥ 3), *n* (%)	40 (11.7)	17 (25)	**0.007**
AKI (AKIN ≥ I), *n* (%)	176 (51.6)	45 (66.2)	**0.033**
Length of hospital stay (days), median (IQR)	6 (5–7)	7 (6–10)	** < 0.001**
Readmission rate, *n* (%)	20 (5.9)	11 (16.2)	**0.009**

Bold font indicates statistical significance

**Fig. 1:**
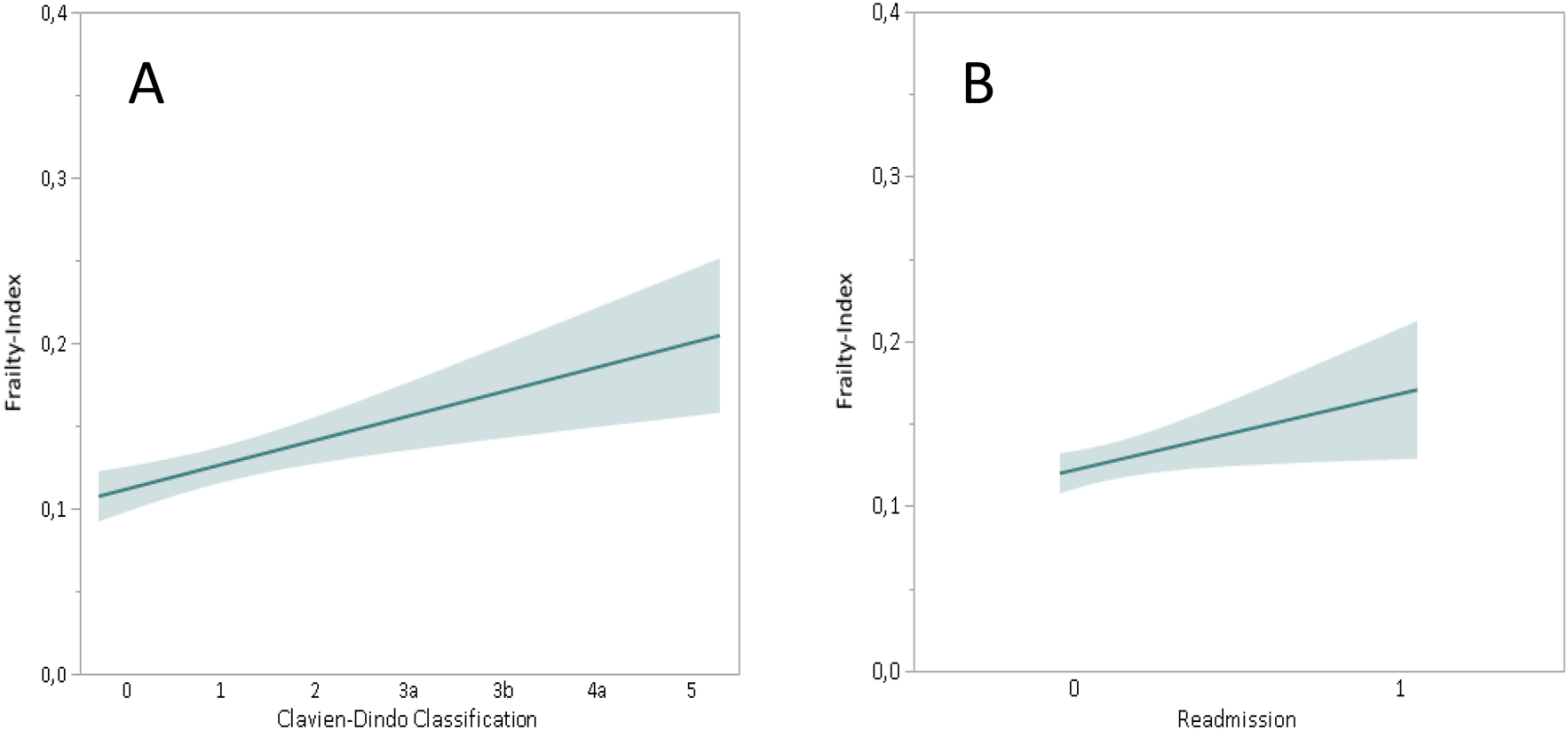
Logistic regression for the 11-CSHA frailty index and the Clavien–Dindo classification (**a**) and the readmission (**b**) for the NSS cohort

Figure 1 illustrates the correlation between the variables frailty (11-CSHA) and complications (Clavien–Dindo classification) and the re-admission to hospital, respectively. The higher the frailty-index, the more severe are the complications and the higher is the probability for a re-admission.

### Uni- and multivariable analyses for severe complications and hospital re-admission

We performed uni- and multivariable analyses for the prediction of severe complications (Clavien–Dindo ≥ 3) and for the prediction of hospital re-admission in the NSS study cohort as given in Tables 4, 5. A solitary kidney was identified as significant predictor for the development of severe complications with an OR of 3.89 (95% CI 1.5–10.03, *p* = 0.005) but not for the need of hospital re-admission.

**Table 4:** Uni- and multivariable analyses for severe complications (Clavien–Dindo ≥ 3) in the NSS cohort

	Univariable analysis			Multivariable analysis		
	Odds ratio	95% CI	*p* value	Odds ratio	95% CI	*p* value
Clavien–Dindo ≥ 3						
Solitary kidney	3.69	1.56–8.76	**0.003**	3.89	1.5–10.03	**0.005**
11-CSHA ≥ 0.27	2.51	1.32–4.76	**0.005**	3.60	1.68–7.72	**0.001**
aaCCI ≥ 6	1.28	0.59–2.78	0.535	0.64	0.24–1.70	0.375
RENAL ≥ 8	2.00	1.13–3.55	**0.018**	2.39	1.29–4.42	**0.005**
Sarcopenia	1.30	0.69–2.45	0.419	0.74	0.38–1.45	0.382
Hypoalbuminemia	0.88	0.35–2.2	0.784	0.93	0.35–2.48	0.892

Bold font indicates statistical significance

**Table 5:** Uni- and multivariable analyses for hospital readmission in the NSS cohort

	Univariable analysis			Multivariable analysis		
	Odds ratio	95% CI	*p* value	Odds ratio	95% CI	*p* value
Hospital readmission						
Solitary kidney	1.02	0.23–4.52	0.982	0.97	0.21–4.55	0.969
11-CSHA ≥ 0.27	3.1	1.41–6.81	**0.005**	1.84	0.68–4.97	0.226
aaCCI ≥ 6	4.28	1.92–9.55	**0.001**	4.93	1.71–14.23	**0.003**
RENAL ≥ 8	2.2	1.03–4.72	**0.043**	2.65	1.18–5.94	**0.018**
Sarcopenia	0.72	0.31–1.66	0.443	0.46	0.19–1.19	0.112
Hypoalbuminemia	1.02	0.29–3.51	0.978	0.58	0.15–2.21	0.428

Bold font indicates statistical significance

The frailty score11-CSHA could be identified as a significant predictor for the development of severe complications with an OR of 3.60 (95% CI 1.68–7.72, *p = *0.001) but not for the necessity of hospital re-admission. Contrary to this, the aaCCI showed significant results for hospital re-admission (OR = 4.93, 95% CI 1.71–14.23, *p = *0.003) but not for the development of severe complications. Tumor complexity (RENAL ≥ 8) could be identified as another significant predictor for both endpoints, for the development of severe complications (OR 2.39, 95% CI 1.29–4.42, *p = *0.005) and for the need of hospital re-admission (OR 2.65, 95% CI 1.18–5.94, *p = *0.018).

Sarcopenia and hypoalbuminemia could not be identified as predictors for severe complications or for hospital re-admission after NSS in the uni- and multivariable analyses.

### Uni- and multivariable analyses for AS/TA as treatment option

To determine possible influencing factors on the chosen therapeutic strategy, we performed uni- and multivariable analyses with all NSS patients and the AS/TA cohort (*n *= 447). The frailty and comorbidity scores were summarized in this analysis. If at least one or both scores were positive, we could identify this factor as predictor for rather choosing AS/TA as therapy strategy compared to NSS (95% CI 2.49–33.88, *p* < 0.001). Furthermore, having a solitary kidney showed to be an independent predictor to rather undergo AS/TA with an OR of 5.43 (95% CI 1.65–17.89, *p = *0.005). Another factor influencing the therapy choice was hypoalbuminemia with an OR of 4.6 (95% CI 1.47–14.35, *p = *0.009). Tumor complexity (RENAL ≥ 8), ASA > 2 and sarcopenia did not show to be predictors to rather decide for AS or TA as treatment option than for surgery in the multivariable analysis. In the univariate analysis, 11-CSHA ≥ 0.27 and/or aaCCI ≥ 6, a solitary kidney, ASA > 2 and hypoalbuminemia all showed to be significant factors to rather undergo AS/TA than NSS. The results of the uni- and multivariable analyses are given in Table 6.

**Table 6:** Uni- and multivariable analyses for AS/TA as treatment option

	Univariable analysis			Multivariable analysis		
	Odds ratio	95% CI	*p* value	Odds ratio	95% CI	*p* value
Active surveillance/tumor ablation						
11-CSHA ≥ 0.27 and/or aaCCI ≥ 6	13.1	5.81–29.57	** < 0.001**	9.19	2.49–33.88	** < 0.001**
Solitary kidney	6.0	2.68–13.43	** < 0.001**	5.43	1.65–17.89	**0.005**
RENAL ≥ 8	1.45	0.71–2.92	0.305	1.62	0.52–5.03	0.40
ASA > 2	4.38	2.19–8.77	** < 0.001**	0.91	0.29–2.85	0.87
Sarcopenia	1.06	0.39–2.89	0.906	0.51	0.15–1.69	0.272
Hypoalbuminemia	12.65	6.0–26.68	** < 0.001**	4.60	1.47–14.35	**0.009**

Bold font indicates statistical significance

## Discussion

It is known that frailty puts patients undergoing surgery at a higher risk for developing poor healthcare outcomes [22–24]. Frailty characteristics and geriatric syndromes predict the occurrence of postoperative complications, which could be shown in diverse major surgeries [14, 25]. To operate or to decide for alternative therapeutic strategies is the question faced by surgeons and elderly patients when they present with the indication for major operation. Besides tumor characteristics patient characteristics, such as frailty and comorbidities, seem to be the main aspects determining the postoperative outcome. This points to the question whether patient characteristics have an equivalent relevance and influence as tumor complexity on the postoperative outcome and complications.

In the field of renal masses, we have the advantageous situation that we can offer alternative therapeutic strategies besides NSS, in particular for SRM, which can be interesting especially for older patients. To our knowledge, the relationship between frailty syndrome and patients undergoing NSS was not assessed by now.

In the current study, we analyzed patient and tumor characteristics for their association with postoperative complications and hospital re-admission in patients undergoing NSS. Furthermore, we assessed whether frailty and comorbidities, tumor complexity and other parameters, such as ASA score, single kidney, albumin level or sarcopenia, are determining factors for the therapeutic strategy patients and clinicians choose as treatment for renal masses.

We found that the frailty scores 11-CSHA and aaCCI were significantly associated with either postoperative severe complications (11-CSHA: OR 3.60, *p = *0.001) or hospital re-admission (aaCCI: OR 4.93, *p = *0.003). The 11-CSHA has been explored to facilitate the measurement of frailty based on clinical judgement [14]. The aaCCI was developed for the evaluation of comorbid conditions and quantifies an individual’s burden of disease and is a tool to predict surgical mortality [15].

Frail and comorbid patients needed significantly more blood transfusions compared to non-frail patients (*p = *0.001). Perioperative blood transfusions can affect the oncological outcome adversely in patients undergoing kidney surgery [26]. The effect of perioperative blood transfusion on renal function seems to potentially lead to an additional impairment of the kidney function besides the surgically caused damage [27]. In line with the fact that we observed significantly more severe complications (CDC ≥ 3) in frail patients (*p = *0.007), we could also record a longer hospital stay (*p* < 0.001) and a higher rate of re-admission (*p = *0.09) in those patients compared to non-frail patients. A significant correlation of higher hospital re-admission rates and the 11-CSHA could also be shown in diverse other surgeries concerning frail and non-frail patients [28]. We know about the negative hospitalization outcomes in older adults and the general decline in function in those patients [29, 30]. Thus, the length of the hospital stay should maximally be shortened, especially in frail patients. The rate of postoperative AKI was likewise higher in the frailty cohort compared to non-frail patients (*p = *0.033). There is evidence, that patients with SRM are more likely to suffer and die of complications related to postoperative renal failure than of the cancer itself [31]. Thus, it is of importance to protect especially the predisposed of developing renal failure and to consider alternatives to NSS in vulnerable patients.

Compatible with the result of significantly more severe complications in frail patients, the TRIFECTA criteria were obviously less achieved in frail patients, as urological complications are one of the TRIFECTA criteria (49.9% vs. 36.8%). Interestingly, sarcopenia, which is considered as a key component of frailty, could not be identified as a predictor for severe complications (OR 0.74) or hospital re-admission (OR 0.46) [22, 32]. The same result could be observed for hypoalbuminemia. Although being well known as a marker of frailty, hypoalbuminemia did not show to be a significant predictor for severe complications after NSS (OR 0.93) or for hospital re-admission (OR 0.58) [33, 34]. We assume that both factors themselves are not the most decisive components in the context of the frailty syndrome.

The aaCCI could not be identified as a significant predictor for severe complications in the uni- and multivariable analyses (OR 0.64, *p = *0.375), but for hospital re-admission (OR 4.93, *p = *0.003). Similar results could be shown in other studies examining the correlation between severe complications and radical nephrectomy [35] or prostatectomy [36], respectively [37]. The 11-CSHA showed to be a reliable measuring tool to assess the risk of severe postoperative complications (OR 3.60, *p = *0.001) in patients undergoing NSS. However, the 11-CSHA did not show to have a significant association with hospital readmission in this study (OR 1.84, *p = *0.226). Adversely, in patients who were readmitted to the hospital after falling at home an association with the CSHA could be confirmed [38]. We know that one of the main predictors for postoperative hospital readmissions are post-surgical complications [39]. Thus, one would expect that the 11-CSHA would also be a predictor of hospital readmission. This discrepancy could be explained by the fact that we did not evaluate whether patients were readmitted to other hospitals than ours. Thus, the number of readmissions might be higher than indicated.

Deciding which patient with a renal tumor is unsuitable for surgical intervention but a good candidate for AS or TA is depending on diverse factors. In daily clinical practice, besides solitary kidneys and kidney function, the overall impression of a patient plays an important role in advising the patient to the most suiting therapy option. In the process of decision-making clinical tests are rarely used to evaluate frailty. In our analysis with AS/TA as endpoints, we could identify the aaCCI > 6 or/and 11-CSHA ≥ 0.27 (OR 9.19, *p* < 0.001), single kidney (OR 5.43, *p = *0.005) and hypoalbuminemia (OR 4.60, *p = *0.009) as predictive factors to rather undergo AS or TA as therapy in patients with renal cancer. This underlines the fact that besides tumor anatomy, patient characteristics, such as frailty, play a decisive role concerning the therapy patients receive. The fact that tumor complexity (RENAL ≥ 8) did not show to be a significant influencing factor on choosing one of the therapy options underlines the assumption that even complex tumors should be treated in consideration of the overall medical status of the patient.

The risk to develop perioperative complications plays a crucial role for therapy planning, especially in frail patients. Given the fact that we can offer alternative treatments to NSS patient characteristics in terms of frailty and comorbidities should more carefully be payed attention to. Besides tumor complexity, patient characteristics in terms of frailty parameters and comorbidities seem to play an important role in the postoperative outcome.

Our findings highlight the importance of clinicians assessing frailty parameters and comorbidities in a preoperative setting in older adults to support decision-making and therapy planning in patients presenting with renal masses. Establishing simple frailty assessment tools and incorporating the measurements into clinical practice could help improving medical care for older adults undergoing kidney surgery.

Our study is limited by the retrospective design with inherent biases therein. Furthermore, we performed a single-center study. Therefore, hospital-related characteristics, such as specific internal standards, could influence the outcome. Moreover, there was no data acquisition in terms of polypharmacy, which has already been identified in other studies as a possible independent predictor of postoperative complications [40]. Large study populations give more reliable results with greater precision and power. Our NSS cohort is quite large, however, our AS/TA cohort consisted of a rather small number of patients.

For the assessment of frailty, we used the 11-CSHA and the aaCCI, which is a tool for the measurement of comorbidity. In this regard, the use of multiple validated geriatric assessment tools could help to assess frailty even more precisely. Furthermore, there is a risk of selection bias at treatment choice. Besides regression, more sophisticated methods, such as propensity score matching, can be used to account for baseline differences between groups [41]. This requires a multidisciplinary approach between clinicians and statisticians to carefully select the most appropriate test for the sample at hand.

## Conclusion

In this study, we could show that the frailty and comorbidity status of patients undergoing NSS has an important influence on the postoperative outcome concerning complications and hospital readmission. Additionally, apart from tumor anatomy, frailty and comorbidities seem to be independent predictors to rather undergo AS/TA than surgery. Thus, frailty parameters should preoperative be assessed carefully, especially in elderly patients, and used as basis for therapy planning and decision-making.

## Supplementary Information

Below is the link to the electronic supplementary material.

Supplementary file1 (DOCX 12 KB)
