# Supplementary material for: Frailty predicts outcome of partial nephrectomy and guides treatment decision towards active surveillance and tumor ablation
Source: World J Urol. 2021 Jan 30;39(8):2843–51. doi: 10.1007/s00345-020-03556-7 (PMC8405500; doi:10.1007/s00345-020-03556-7)
Supplement: Supplementary file 1 — Supplementary file1 (DOCX 12 KB) [file 345_2020_3556_MOESM1_ESM.docx]

| **Specific severe complication** | **Clavien-Dindo classification** |
| --- | --- |
| aneurysm requiring embolization (n = 22) | 3a |
| pneumothorax requiring thoracic drainage (n = 20) | 3a |
| revision due to postoperative bleeding (n = 10) | 3b |
| acute renal failure requiring dialysis (n = 7) | 4a |
| haematoma requiring retroperitoneal drainage (n = 3) | 3a |
| urinoma requiring retroperitoneal drainage (n = 1) | 3a |
| acute myocardial infarction requiring stenting (n = 1) | 3b |
| urinoma requiring ureteral stenting (n = 1) | 3b |
| wound dehiscence requiring revision (n = 1) | 3b |
| death due to septic shock, colon ischemia, multi-organ failure (n = 1) | 5 |
| death due to septic shock, multi-organ failure (n = 1) | 5 |
